# NK Cell Subtypes as Regulators of Autoimmune Liver Disease

**DOI:** 10.1155/2016/6903496

**Published:** 2016-07-04

**Authors:** Guohui Jiao, Bangmao Wang

**Affiliations:** Department of Gastroenterology, General Hospital, Tianjin Medical University, Tianjin 300052, China

## Abstract

As major components of innate immunity, NK cells not only exert cell-mediated cytotoxicity to destroy tumors or infected cells, but also act to regulate the functions of other cells in the immune system by secreting cytokines and chemokines. Thus, NK cells provide surveillance in the early defense against viruses, intracellular bacteria, and cancer cells. However, the effecter function of NK cells must be exquisitely controlled to prevent inadvertent attack against normal “self” cells. In an organ such as the liver, where the distinction between immunotolerance and immune defense against routinely processed pathogens is critical, the plethora of NK cells has a unique role in the maintenance of homeostasis. Once self-tolerance is broken, autoimmune liver disease resulted. NK cells act as a “two-edged weapon” and even play opposite roles with both regulatory and inducer activities in the hepatic environment. That is, NK cells act not only to produce inflammatory cytokines and chemokines, but also to alter the proliferation and activation of associated lymphocytes. However, the precise regulatory mechanisms at work in autoimmune liver diseases remain to be identified. In this review, we focus on recent research with NK cells and their potential role in the development of autoimmune liver disease.

## 1. Introduction

Natural killer (NK) cells are important effectors of innate immunity, constituting up to 15% of the lymphocytes in human peripheral blood. Additionally, in several tissues and organs, their numbers are highly enriched [1]. NK cells are mainly involved in the defense against early viral infection, intracellular bacteria, and cancer cells [2]. By secretion of cytokines and chemokines [3], NK cells have the ability to interact with other immune cells to orchestrate immune responses linking innate immunity with adaptive immunity, that is, priming, influencing, and regulating T cells, B cells, and dendritic cells (DCs) [4]. NK cells express multiple activating and inhibitory receptors on their surfaces, and these receptors are the sites where ligands on target cells attach and control the dynamic signal balance [5, 6].

The liver is constantly exposed to food-derived antigens, microbes, and molecules absorbed into the intestinal system from the gastrointestinal tract via the portal vein. The liver is crucial to maintaining immune tolerance and to providing a correct defense against pathogens [7].

NK cells as innate immune cells physiologically resident in the liver must constantly participate in the maintenance of balance. Otherwise, inflammatory or autoimmune liver disease develops. Recently, accumulating data have demonstrated that NK cells play important but opposite roles involving the tissue cells as their targets at various stages of a corresponding autoimmune disease, such as that in the liver [8]. Other advances have significantly shaped the understanding of NK cell biology, broadening our appreciation of their influence on the immune system and the exquisite regulation of the immune balance.

This review focuses on recent research linking NK cells with autoimmune liver diseases, particularly the regulatory function of NK cells in maintaining homeostasis and their potential role in therapeutic applications.

## 2. Natural Development of NK Cells

Peripheral blood NK (PB-NK) cells were identified as empowered to lyse “nonself” cellular targets, an action controlled by inhibitory NK receptors (iNKRs) [9]. Activating NK receptors and coreceptors that trigger cytolytic activity include the natural cytotoxicity receptors (NCRs) NKp46, NKp30, and NKp44. Activating forms of lectin-type receptors such as NKG2C or NKG2D or killer cell immunoglobulin-like receptors (KIRs) are also expressed [10] and facilitate the early phase response without immunological memory [11, 12].

NK cells were officially classified as the prototypical members of the group 1 innate lymphoid cells, which are defined by their capacity to secrete interferon- (IFN-)*γ* [13, 14]. In human, NK cells are classically defined as CD56^+^CD3^−^ cells [15] and occupy primarily the blood, spleen, liver, lung, and bone marrow, although limited numbers are localized in lymph nodes [16].

The two major subsets of NK cells found in humans are CD56^dim^ and CD56^bright^. CD56^dim^ NK cells are fully mature, make up approximately 90% of the NK cells in peripheral blood, and mediate cytotoxicity responses. In contrast, CD56^bright^ cells are relatively immature, making up approximately 5% to 15% of total NK cells and considered primarily as cytokine producers in lymph nodes [17, 18]. The CD56^bright^CD16^−^ subset is believed to manifest as suppressors of the self-reactive T cell response and inducers of apoptosis in activated T cells [19, 20].

NK cells are a major source of the type 1 cytokine IFN-*γ*, as well as tumor necrosis factor (TNF), granulocyte-macrophage colony-stimulating factor (GM-CSF), other cytokines, and chemokines [12]. These soluble factors have important regulatory influences over the recruitment and function of multiple immune cell populations [21].

NK cells are central players in a regulatory crosstalk network in the context of immunological responses against inflammatory stimuli [22]. In this regard, NK cells engage in active and bidirectional communications with autologous DCs requiring that both cell types interact and secrete specific cytokines [23]. Additionally, NK cells are able to interact with monocytes and macrophages [24, 25]. Furthermore, the cytotoxic function of NK cells is mediated by the directed exocytosis of cytolytic granules to release perforins and granzymes, respectively, perforating the target cells' plasma membrane and triggering apoptosis.

## 3. NK Cell Distribution and Liver Microenvironment

The liver is now increasingly regarded as the largest organ of innate immunity enriched as it is in cells with innate immune properties [26]. In fact, NK cells make up as many as 30–50% of hepatic lymphocytes recruited by the liver's microenvironment [27, 28]. Hepatic NK cells are extremely sensitive to the activation of interleukin- (IL-) 2, which is associated with IL-2-mediated upregulation of tumor necrosis factor-related apoptosis-inducing ligand (TRAIL) [29].

**Figure 1:**
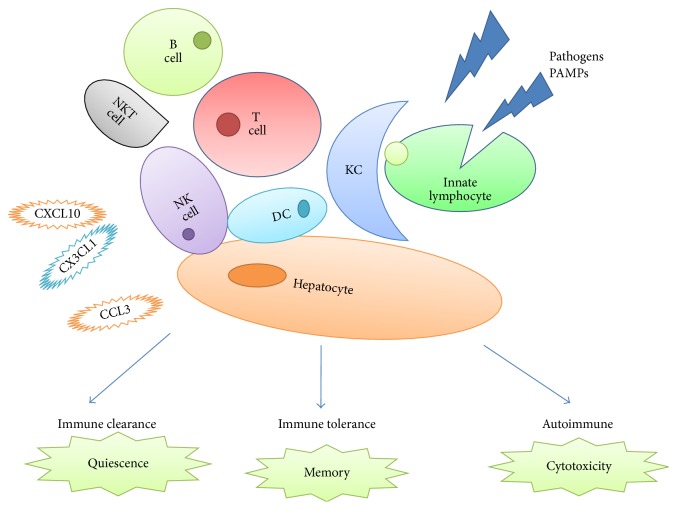
NK cells interaction with other immune cells in liver autoimmunity. Pathogen associated molecular patterns (PAMPs) can activate innate immunity, especially the innate lymphocytes (NK, ILC subsets, NKT, and *γδ*T lymphocytes). This can be regulated by reciprocal interactions among stromal cells, monocytes, and dendritic cells. Innate response elicited by NK leads to (a) rapid elimination of the pathogens; (b) triggering generation of memory T and B cells; and (c) breaking the tolerance by chronic stimulation leading to generation of autoreactive cytotoxicity (KC: Kupffer cell; NKT: natural killer T; DC: dendritic cell).

The liver receives blood from the portal vein, which contains products of digestion and antigens or microbial elements from intestine, but hepatic lymphocytes do not respond to these components at physiological status. Accordingly, Wu et al. have recently found that hepatic NK cells were kept in an immature state within the liver's microenvironment [30]. However, unlicensed NK cells can be mobilized and activated in response to inflammatory signals, after which an autoimmune response may occur [31, 32] (Figure 1).

NK cells also display potent regulatory effects on innate and adaptive immunity [33, 34]. For example, they provide signals promoting DC function and T helper cell polarization. NK cells also interact with immature DCs or autoreactive T cells to maintain immune homeostasis [35]. Previous work revealed that the liver's microenvironment influences the unique phenotype and development of liver NK cell subsets [36]. NK cell receptors can become phenotypically modified so as to promote high levels of the inhibitory receptor NKG2A, while losing expression of MHC class I-binding Ly49 receptors, thus sustaining the functional hyporesponsive status of NK cells. This effect depends on the high levels of IL-10 within the liver [37]. A novel NK subpopulation characterized by CD25, CD93, and CX3CR1 expression, but near absence of CD62L, CD11b, and CD27, exerts potent cytolytic activity and abundant IFN-*γ* production. However, the role of this novel subset in physiological normality and in the pathogenic process of autoimmune diseases needs further clarification [38].

Hepatic NK cells could retain antigen-specific memory against haptens and virus-derived antigens, crucially dependent on the expression of CXCR6 in order to reside in sinusoidal spaces to protect the liver from NK cell-mediated hepatotoxicity [39]. Questions remain regarding the mechanistic foundations for these memory responses, how long they can be maintained, and whether they can be harnessed to combat disease through therapeutic interventions, such as cell-based strategies.

## 4. Role of NK Cells in Autoimmune Liver Diseases

Primary biliary cirrhosis (PBC), autoimmune hepatitis (AIH), and primary sclerosing cholangitis (PSC) are the three major forms of autoimmune liver disease. Each has a unique pattern of inflammation, clinical phenotype, and focus of autoimmune injury [40, 41] (Table 1).

### 4.1. NK Cells in PBC

Currently, autoreactive B cells and burgeoning numbers of T cells are believed to attack and destroy the small intrahepatic bile ducts in PBC disorder. Growing evidence suggests that patients with PBC manifest increases in both the frequency and absolute number of PB-NK cells at the systemic and local levels [42] and that such patients express abnormally high levels of perforin along with decreases in cytokines [43, 44].

Chuang et al. [45] reported a clearly higher frequency and absolute number of NK cells in both the blood and liver of PBC patients, along with elevated cytotoxic activity, perforin expression, and levels of plasma IL-8, with marked expression of IL-8R on such cells. A TRAIL-dependent mechanism is crucial for the NK cell-mediated lysis of biliary epithelial cells and results in cholestatic liver injury [46, 47].

Within the livers of PBC patients, CD56^dim^/CD16^pos^ cell infiltration was obvious [48] with strong cytotoxic activity against autologous biliary epithelial cells [49, 50]. Increased numbers of CD56^+^ cells had scattered around the destroyed small bile ducts. This pathogenic effect required a crosstalk between Toll-like receptors (TLR) and NK receptors. Recently, Shimoda et al. [49] demonstrated that TLR4 ligand-stimulated NK cells destroyed autologous BECs (biliary epithelial cells) in the presence of IFN-*α* synthesized by TLR3 ligand-stimulated monocytes.

Chemokines play an important role in destruction of the biliary tract [51, 52] by recruiting cells of the immune system. As such, NK cells have been reported to express the chemokine receptors CX3CR1 and CXCR3 [53]. A forward-thinking hypothesis of PBC's etiology is that the increased migration of NK cells to liver is chemokine receptor-dependent, thus breaking NK cell immune tolerance [54].

### 4.2. NK Cells in AIH

T lymphocytes have been reported to play a prominent role in the pathogenesis of AIH, although the participation of innate immune cells, such as NK cells, has also been confirmed [55]. In mice, the administration of poly I:C, an analogue of double-stranded RNA, generated NK cell-dependent hepatitis and triggered pathological role of IL-17 as in AIH [56]. Killer immunoglobulin-like receptors are key regulators of natural killer cell-mediated immune responses. NK cells with their key receptors KIR gene KIR2DS1 were important in AIH genesis with high affinity HLA-C2 ligands, contributing to unwanted NK cell autoreactivity in AIH-1 [57].

### 4.3. NK Cells in PSC

PSC has been consistently associated with the presence of certain HLA alleles, yet the etiopathogenesis of this disease is virtually unknown [58]. However, genetic variation of the NKG2D receptor has been linked with development of cholangiocarcinoma in PSC patients [59, 60]. Additionally, NK cells in the peripheral blood and in the colonic mucosa could be observed during the course of PSC [61]. Liver NK cells from PSC patients have a decreased cytolytic activity likely due to the high levels of local tumor necrosis factor- (TNF-)*α* production [62].

## 5. Role of NK Cells in Autoimmune Regulation

With its functional alteration and inhibition of both proliferation and activation of autoreactive T lymphocytes, macrophages, and DCs, NK cells have assumed a regulatory function with respect to available quantities of inflammatory cytokines. For example, poly I:C-activated NK cells exhibited a protective role in a model of Con A-induced hepatitis by suppressing T or NKT cell activation [63]. Cytokines are believed to be crucial for NK cell function. However, the neutralization of IL-17A by monoclonal antibodies reduced the accumulation, activation, and cytolytic activity of poly I:C-induced intrahepatic NK cells [56]. Another liver injury factor, 2,3,7,8-tetrachlorodibenzo-p-dioxin (TCDD), promoted IFN-*γ* induction by NK cells, thereby exacerbating the immune-mediated liver injury induced by Con A [64]. Under the attractive effect of chemokines, NK cells infiltrated the liver and interacted with stromal cells [65]. Moreover, CCL2, CCL3, and CXC-chemokine ligand 10 (CXCL10) can recruit NK cells expressing CCR1, CCR2, CCR3, CCR5, and CXCR3 to the liver.

More importantly, NK subsets may behave differently in diverse tissue and target organs. Depending on the opportunity for crosstalk with organ-resident cells, together with the specific effect of various cytokine microenvironments, NK cell function is shaped by cell surface receptors or membrane molecules. NK cells might lyse antigen-presenting cells in the T cell areas of lymphoid organs, inhibiting signals for T cell activation. Further, through production of IFN-*γ*, NK cells perform multidirectional regulation. T cells could even stimulate NK cell activation under the influence of IL-2 [66].

Immune checkpoint molecules blockers are widely recognized as therapeutic agents in oncology, which could also arouse immune-related adverse events including hepatitis [67]. KIRs that deliver inhibitory signals to NK cells might contribute to preventing unwarranted innate immune responses against healthy cells, which could play a role in the above-mentioned event [68]. Another costimulatory receptor 4-1BB agonist could modulate the NK cells activity. It was reported to have the potential of antitumor and antiviral immunity, while, unlike other immune checkpoint molecules blockers, ameliorating autoimmune disease. However, 4-1BB agonists can trigger high grade liver inflammation. The contribution of NK cells in the phenomenon needs to be further elucidated [69].

Malfunction of NK cells is closely related to liver malignancies. Genetic variation of natural killer cell receptor G2D (NKG2D) is associated with development of CCA in PSC patients [59]. Alteration of collagen-binding integrins expression in liver tissue-resident NK cells led to defect in NK maturation contributing to tumor genesis [70]. Further studies are needed to demonstrate the role of NK cells in autoimmune liver disease-related carcinogenesis.

## 6. Conclusion

Considerable evidence has shown that NK cells are major players in mediating the pathogenesis of autoimmunity. This concept provides an explanation for the unsatisfactory therapeutic effect of ordinary immune-suppression techniques [71]. In view of the influence of local microenvironments on the behavior and function of NK cells, NK cell dysfunctions should be regarded as epiphenomena determined by the presence of autoreactive B and T cells. The liver, with its constant exposure to external and non-self-antigenic elements from gut and lymph circulation, represents a unique microenvironment, especially prone to the development of autoimmune disease. Unquestionably, fuller understanding of NK cell self-tolerance and autoreactivity regulation in the liver offers enormous potential as a foundation for the development of new, broadly applicable immune-based therapeutics.

## Figures and Tables

**Table 1:** Roles of NK cell in the pathogenesis of autoimmune liver diseases.

Disease	NK cell characteristics	Roles of NK cell in the pathogenesis of disease	Reference
PBC	CD56^dim^/CD16^pos^ CX3CR1^+^ CXCR3^+^	(1) Elevated cytotoxic activity, perforin expression, and levels of plasma IL-8 (2) Increased TRAIL expression as an apoptotic inducer in portal tract damage (3) With TLR4 ligand-stimulated NK cells	[45–49, 53, 54]

AIH	CD16^+^CD56^+^ CD69^+^, CCR5^+^ CXCR6^+^	(1) Migration in response to the chemotactic stimuli (2) KIR gene KIR2DS1 in AIH genesis with high affinity HLA-C2 ligands	[39, 57]

PSC	CD16^+^CD56^+^	(1) Decreased cytolytic activity (2) NKG2D associated with development of CCA	[58–61]
